# Pernicious Anemia With Spuriously Normal B12 Levels

**DOI:** 10.7759/cureus.48937

**Published:** 2023-11-17

**Authors:** Ana Isabel Brochado, Joana Marques Dias, Marta Azevedo Ferreira, Ana Grilo, Ana Gonçalves

**Affiliations:** 1 Internal Medicine Department, Hospital Beatriz Ângelo, Loures, PRT; 2 Intensive Care Unit Department, Hospital Beatriz Ângelo, Loures, PRT

**Keywords:** megaloblastic anemia, hemolysis, vitamin b12, pancytopenia, pernicious anemia

## Abstract

We present a 29-year-old man admitted to our hospital with fatigue for two months of duration and recent palpitations, lightheadedness, blurred vision and nausea. Workup showed pancytopenia with severe macrocytic anemia, laboratory and blood smear features of hemolysis, low reticulocyte percentage and a negative direct Coombs test. B12 and folate levels were normal. As bone marrow aspirate was suggestive of megaloblastic anemia and upper endoscopy showed atrophic gastritis, we ordered homocysteine (elevated) and intrinsic factor (IF) antibodies (positive). The workup led to the diagnosis of pernicious anemia with spuriously normal B12 levels. Replacement therapy allowed a rapid recovery. We highlight that the presence of IF antibodies can interfere with the competitive binding assays commonly used to measure B12 levels.

## Introduction

Pernicious anemia (PA) is an autoimmune disorder that leads to chronic atrophic gastritis and cobalamin (B12) deficiency. It is the leading cause of cobalamin deficiency worldwide.

Its mechanisms are related to the presence of autoantibodies against intrinsic factor (IF) or gastric parietal cells, which lead to the malabsorption of dietary cobalamin [1].

Clinically, PA presents with symptoms of anemia that may be subtle due to its insidious development. Patients can also present with diverse neurologic symptoms, the most classic being paresthesia, balance difficulties and spasticity due to nerve damage in the peripheral nerves, lateral and posterior columns of the spinal cord or in the cerebellum. Other nonspecific neuropsychiatric symptoms such as psychosis, mood and personality changes, and dementia are also associated with cobalamin deficiency [1].

The diagnosis of PA is easily suspected in patients with megaloblastic anemia and cobalamin deficiency. However, there are some clinical scenarios in which the diagnosis is more challenging and, therefore, delayed. PA can present with normal or even elevated cobalamin levels because of the interaction of IF antibodies with the reagents used in the competitive chemiluminescence assays used to measure cobalamin [1,2].

PA can also present with macrocytosis without anemia, pancytopenia, neurological symptoms without anemia or macrocytosis, hemolysis, and even with features of myelodysplastic syndromes of thrombotic microangiopathy [3-6].

We present a case of PA with atypical clinical and diagnostic features.

## Case presentation

A 29-year-old man of African descent presented with generalized fatigue for two months of duration. This fatigue worsened in the days prior to admission with lightheadedness, palpitations, blurred vision and nausea.

He had a medical history of a traumatic cerebral venous thrombosis two years before that was treated with anticoagulation for six months, and macrocytic anemia in the previous year with normal cobalamin and folic acid levels.

At admission, his vitals and observation were unremarkable except for pale mucous membranes.

The initial blood tests showed pancytopenia with severe macrocytic anemia, elevated ferritin and lactate dehydrogenase (LDH), with low haptoglobin, as seen in Table 1. Thyroid levels were normal. The initial clinical scenario pointed to hemolysis but without an adequate bone marrow response. Glucose-6-phosphate dehydrogenase (G6PD) level was also normal. Renal, coagulation and hepatic function tests were normal except for elevated total bilirubin (1.43 mg/dL) due to unconjugated bilirubin. The direct Coombs test was negative. The peripheral smear showed marked anisocytosis and poikilocytosis, with many schistocytes. Viral markers for human immunodeficiency virus (HIV), Hepatitis B (HBV) and C (HCV), Cytomegalovirus (CMV), Epstein-Barr virus (EBV) and Parvovirus B19 were made, and all were negative.

**Table 1:** Laboratory findings at admission and after cobalamin replacement MCV: Mean corpuscular volume, LDH: Lactate dehydrogenase

	Initial workup (unit)	After B12 replacement (unit)	Reference range (units)
Hemogloblin	5.9 g/dL	12.8 g/dL	13.5-17.5 g/dL
Hematocrit	16%	39%	41-53%
MCV	105 fL	93 fL	800-100 fL
Reticulocyte percentage	0.9%	1.2%	0.5-1.5%
Leucocytes	3.29x10^9/L	5.14x10^9/L	4-10x10^9/L
Platelets	84x10^9/L	246x10^9/L	150-400x10^9/L
Serum iron	192 ug/dL	81 ug/dL	33-193 ug/dL
Ferritin	519 ug/L	130 ug/L	30-400 ug/L
Haptoglobin	<8mg/dL	46 mg/dL	30-200 mg/dL
LDH	>5000 UI/L	200 UI/L	132-225 UI/L
Vitamin B12	244 ng/L	692 ng/L	211-911 ng/L
Folate	11.7 ng/mL	7.4 ng/mL	>5.4 ng/mL

Initially, corticosteroids were initiated (three days of methylprednisolone 1g/day), without improvement of the hemoglobin or platelet counts.

Blood marrow aspirate revealed a hypercellular megaloblastic marrow, suggesting a megaloblastic anemia. Upper gastrointestinal endoscopy showed atrophic gastritis. Since B12 deficiency was still suspected, even with normal B12 levels, serum homocysteine was ordered and elevated with 66 umol/L (normal range: 5.5-16.2 umol/L). Antibodies against IF were positive with 186 AU/mL (normal range: 1.21-1.52 AU/mL).

Cobalamin replacement therapy was initiated and led to a fast medullary response with the reticulocyte percent rising within five days and improvement of platelet count and LDH. As seen in Table 1, at reevaluation after two months of therapy, the hemoglobin was 12.8 g/dL with mean corpuscular volume (MCV) 93 fL, reticulocyte percent of 1.2%, resolution of the thrombocytopenia and normalization of LDH and haptoglobin. The patient was asymptomatic.

## Discussion

This case presents an atypical presentation of PA, with normal levels of B12 on competitive chemiluminescence assay and with components of hemolysis, all leading to a need for a higher suspicion to make the diagnosis.

The most important caveat in this is the possibility of failure of the most commonly used assays for the detection of cobalamin deficiency in the presence of IF autoantibodies. Most routine laboratories measure cobalamin levels with competitive binding assays because this technique can be easily automated. However, in the presence of IF autoantibodies, 33% to 53% of samples can have false normal results [7].

Also, clinicians should be aware of the varied possible hematological manifestations of B12 deficiency. In this case, it presented with features of bone marrow failure with hemolysis and a low reticulocyte count - due to intramedullary hemolysis (ineffective erythropoiesis). This, alongside the spuriously normal B12 levels, made the diagnosis harder and even accounted for the prescription of unhelpful therapies.

In cases of macrocytic anemia, pancytopenia/bone marrow failure, anemia with neurological symptoms, hemolytic anemia and even in scenarios suggestive of myelodysplastic syndrome, if the B12 levels are normal or even elevated, clinicians should keep in mind that measuring homocysteine, methylmalonic acid and IF autoantibodies can be useful. This can allow for a faster diagnosis and more rapid resolution, obviating further investigations and even futile treatments.

## Conclusions

To conclude, this case report shows us an atypical case of PA, presenting with pancytopenia, hemolytic characteristics, negative direct Coombs and spuriously normal B12 levels. This initial result led to a delayed diagnosis and to the prescription of unhelpful therapies. With the suspicion of B12 deficiency in mind, an analysis of IF autoantibodies was made and came back positive, confirming the diagnosis. Complete resolution of symptoms and laboratory alterations was possible after initiating cobalamin replacement therapy, until the present day.

The presence of IF autoantibodies can influence the measurement of cobalamin levels in routine laboratories, giving falsely normal results. Healthcare workers should be aware of this and if the clinical suspicion is consistent, measuring other laboratory parameters such as homocysteine, methylmalonic acid and IF autoantibodies can obviate further unnecessary investigations.
